# Disposable vs. conventional NPPV in acute respiratory acidosis

**DOI:** 10.1515/med-2026-1563

**Published:** 2026-11-02

**Authors:** Ertugrul Demir, Meltem Songur Kodik, Ozlem Inci, Ilhan Uz, Mehmet Birkan Korgan

**Affiliations:** Department of Emergency Medicine, Ege University Faculty of Medicine, Izmir, Türkiye; Department of Emergency Medicine, Dokuz Eylul University Faculty of Medicine, Izmir, Türkiye

**Keywords:** acidosis, respiratory, noninvasive ventilation, emergency service, hospital, continuous positive airway pressure, ventilators, mechanical

## Abstract

**Objectives:**

Acute respiratory acidosis requires ventilatory support. Noninvasive positive pressure ventilation (NPPV) may be delayed in crowded emergency settings. Disposable systems may offer advantages, but evidence remains limited.

**Methods:**

This prospective, single-center observational study included adults with acute respiratory acidosis requiring NPPV from September 2024 to April 2025. Patients received conventional ventilator-based NPPV or a disposable oxygen-driven bilevel CPAP/NPPV system according to clinician decision. The primary outcome was 60-min arterial pH; secondary outcomes included physiological responses, intubation, and 24-h mortality.

**Results:**

Of 323 screened patients, 204 were analyzed: 100 conventional and 104 disposable-system patients. After adjustment for baseline imbalances, 60-min arterial pH did not differ significantly between groups; HC3 robust-standard-error and BCa-bootstrap sensitivity analyses gave the same inference. Intubation and 24-h mortality were similar. Exploratory longitudinal analyses suggested improvement in pH and PaCO_2_, but these findings were hypothesis-generating.

**Conclusions:**

Because device allocation was not randomized and the groups differed at baseline in disease-severity variables, these data do not support a reliable efficacy comparison between the two systems and should not be interpreted as evidence of equivalence or superiority. Larger randomized or rigorously controlled studies are needed to clarify the role of disposable noninvasive ventilation in acute hypercapnic respiratory acidosis.

## Introduction

Acute respiratory acidosis is a severe form of hypercapnic respiratory failure associated with high morbidity and adverse clinical outcomes, necessitating prompt diagnosis and early treatment in the emergency department (ED). It often arises from conditions such as chronic obstructive pulmonary disease (COPD) exacerbations, acute cardiogenic pulmonary edema, asthma, pneumonia, and neuromuscular disorders [1]. In patients with severe acidemia and pronounced hypercapnia, delaying ventilatory support is associated with a higher risk of intubation and increased mortality [2].

Noninvasive positive pressure ventilation (NPPV) is a cornerstone of standard care in managing acute respiratory acidosis. When initiated early in appropriately selected patients, NPPV improves alveolar ventilation, corrects acid-base imbalance, reduces partial pressure of carbon dioxide (PaCO_2_) and work of breathing. Additionally, it may lower the need for intubation and improve short-term clinical outcomes [3]. International guidelines recommend NPPV as a first-line ventilatory support strategy for acute hypercapnic respiratory failure [4], [5], [6]. However, the use of conventional mechanical ventilators may delay the timely initiation of NPPV in crowded ED settings. This delay can be attributed to issues such as device availability, setup complexity, and the requirement for experienced personnel [7].

Recent years have seen growing interest in disposable, portable NPPV systems developed for use in the ED and prehospital settings. Among these, the Flow-Safe II+^®^ Disposable BiLEVEL/CPAP system (Mercury Medical, USA) offers several practical advantages, including rapid setup, portability, and operation with a standard oxygen source. However, the effectiveness of these newer-generation systems in hypercapnic respiratory acidosis remains insufficiently established [8], [9], [10].

Therefore, the present study was designed to address this evidence gap by comparing early physiological responses between a disposable NPPV system and conventional ventilator-based NPPV in adults with acute respiratory acidosis treated in the emergency department. The main research question was whether the disposable system provides comparable early improvement in arterial pH and related physiological parameters during the first 60 min of treatment. The study was designed to assess early physiological response rather than time to treatment initiation, which was not measured.

## Methods

### Study design and setting

This prospective, single-center observational comparative study was conducted in the ED of a tertiary care center, Ege University Hospital, in Izmir, Turkey, between September 3, 2024, and April 14, 2025. Adult patients with acute respiratory acidosis requiring NPPV were consecutively screened based on predefined clinical and arterial blood gas criteria.

Sample size was calculated *a priori* based on the expected between-group difference in early arterial pH response from baseline to 60 min, with a minimum required sample of 126 patients assuming a moderate effect size (Cohen’s d=0.50), an alpha level of 0.05, and 80 % power. Enrollment was continued beyond the *a priori* minimum sample size to increase precision. The final analytic sample included 204 patients. The study was conducted in accordance with the STROBE guidelines [11]. This study was conducted in accordance with the Declaration of Helsinki (as revised in 2013) and was approved by the Ege University Faculty of Medicine Clinical Research Ethics Committee (approval no. 24-3/77, March 5, 2024). Informed consent was obtained from all individuals included in this study, or their legal guardians or wards. The primary outcome was the adjusted between-group comparison of arterial pH at 60 min after initiation of NPPV; longitudinal physiological analyses were interpreted as secondary or exploratory. The null hypothesis was that 60-min arterial pH would not differ between device groups. The study was powered to detect a moderate between-group difference and was not designed as an equivalence or non-inferiority trial; accordingly, a non-significant result cannot be interpreted as demonstrating equivalence.

### Study population

Patients aged ≥18 years with acute respiratory acidosis, defined as PaCO_2_ >45 mmHg and pH ≤7.35 on arterial blood gas analysis, were eligible. NPPV was considered in cooperative, hemodynamically stable patients with hypercapnic acidemia and signs of increased work of breathing who were able to protect the airway. These criteria were applied according to routine ED clinical practice and were not protocolized. Patients requiring immediate intubation, those unsuitable for NPPV, and those with confirmed pregnancy were excluded at screening, before NPPV initiation and device selection. Patients who had been started on NPPV but had insufficient baseline or 60-min data were excluded at the analysis stage, after device selection; their distribution by device group is reported in the Results and Figure 2. No imputation was performed, and complete-case analysis was used.

### Ventilation strategy

As this was an observational study, device selection was not determined by the investigators; no randomization or matching was performed. The ventilation device was selected by the treating clinician according to clinical judgment and device availability in the ED. Patients were managed with NPPV using either conventional NPPV (cNPPV) or a disposable NPPV system. In our clinic, Flow-Safe II+ is available as a disposable NPPV system and is routinely used in patients when clinically indicated (Figure 1). Therefore, the disposable NPPV group consisted of patients treated with Flow-Safe II+. In the cNPPV group, ventilatory support was delivered using standard hospital ventilators capable of noninvasive ventilation (Dräger Evita V300 and Mindray SV300). Pressure-targeted noninvasive modes providing bilevel positive airway pressure support were used, and pressure settings were individualized according to clinical status, respiratory effort, tolerance, oxygenation, and arterial blood gas findings. In the Flow-Safe II+ group, pressure levels were adjusted via the integrated manometer according to oxygen flow and patient response. Initial inspiratory and expiratory pressure settings at NPPV initiation were recorded for each participant. Subsequent changes in ventilator settings during the first 60 min were not systematically captured. FiO_2_, oxygen flow rate, mask interface type, and the interval from ED arrival to NPPV initiation were not systematically recorded.

**Figure 1:**
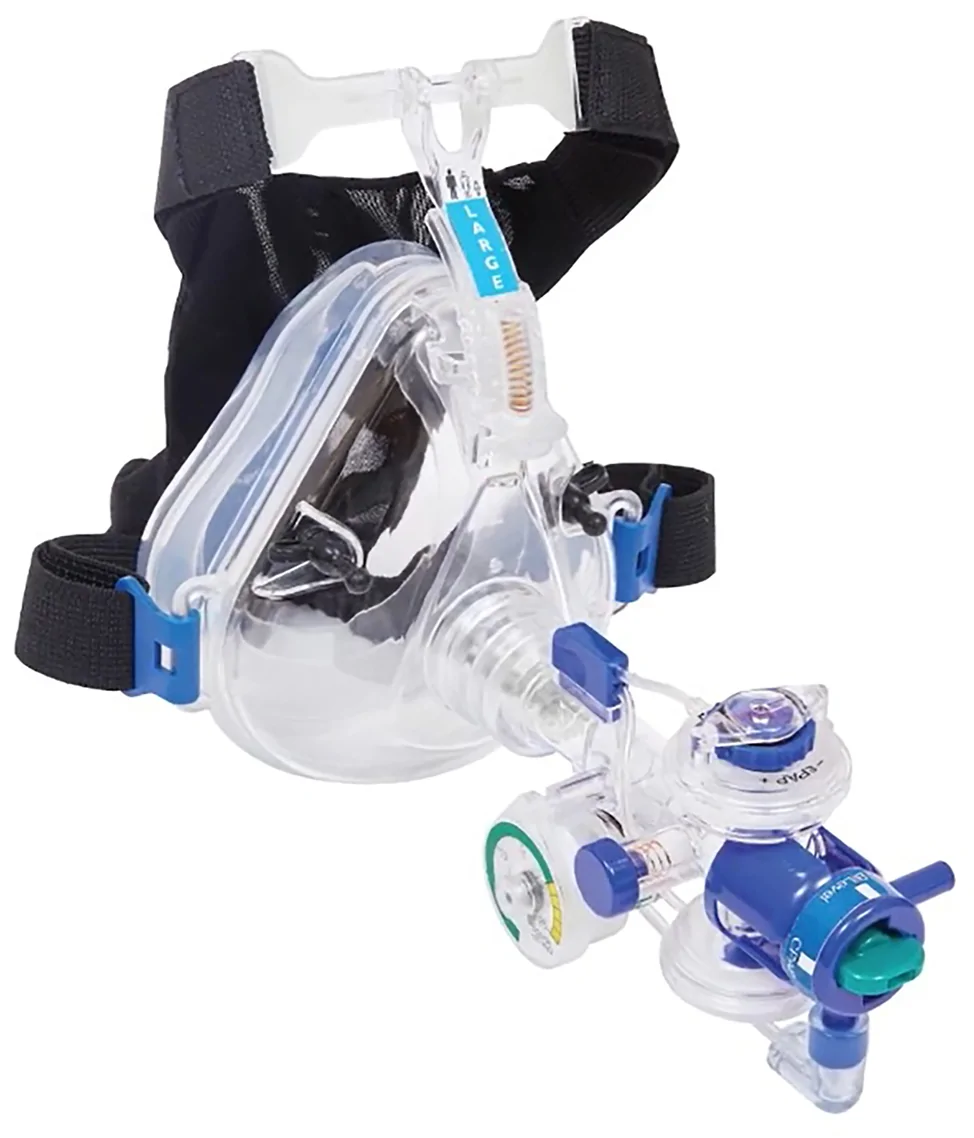
The Flow-Safe II+^®^ disposable BiLEVEL/CPAP system (Mercury Medical, Clearwater, FL, USA), showing the full-face mask with adjustable head strap, integrated manometer, and IPAP/EPAP regulator valves. The device is operated using a standard oxygen source and is designed for rapid setup in emergency and prehospital settings.

### Data collection and outcomes

At presentation (baseline) and 60 min after initiation of NPPV, demographic characteristics, comorbidities, vital signs [systolic blood pressure (SBP), diastolic blood pressure (DBP), heart rate (HR), respiratory rate (RR), and oxygen saturation (SpO_2_)], clinical examination findings [orthopnea, tachypnea, use of accessory respiratory muscles, and Glasgow Coma Scale (GCS) score], and arterial blood gas parameters [pH, PaCO_2_, bicarbonate (HCO_3_^−^), base excess (BE), and lactate] were recorded. The analytic dataset also contained the initial inspiratory and expiratory pressure settings (cmH_2_O) at NPPV initiation for each participant. The baseline arterial blood gas was obtained on arrival, before initiation of NPPV, and was the sample that established eligibility and prompted the decision to start noninvasive ventilation. The interval between this sample and the initiation of NPPV was not recorded, and follow-up measurements were obtained 60 min after initiation. Data were prospectively collected using standardized case report forms by emergency medicine residents and senior physicians who were not involved in the final statistical analysis, and all data were anonymized before analysis.

The primary outcome was arterial pH at 60 min after initiation of NPPV, analyzed with adjustment for baseline arterial pH and clinically selected baseline covariates. This approach was chosen in preference to analysis of the simple change score because baseline adjustment provides greater statistical efficiency and reduces the influence of baseline imbalances on the estimated between-group difference. The choice of 60 min reflected the typical interval at which early physiological response to NPPV is evaluated in emergency department practice. Secondary outcomes were categorized as physiological and clinical outcomes. Physiological secondary outcomes included changes in arterial PaCO_2_, HCO_3_^−^, lactate, BE, HR, SBP, DBP, RR, SpO_2_, and GCS during the first 60 min. Clinical secondary outcomes included intubation rate and 24-h mortality.

### Statistical analysis

Due to the observational design and non-randomized device selection, selection bias and confounding by indication cannot be excluded. Baseline balance between groups was assessed using standardized mean differences (SMD), with values <0.20 considered indicative of no meaningful imbalance, and potential confounders were addressed in multivariable analyses. Statistical analyses were performed using IBM SPSS Statistics version 25.0 (IBM Corp., Armonk, NY, USA) and R version 4.3.2 (R Foundation for Statistical Computing, Vienna, Austria), with the nparLD package used for Brunner–Langer longitudinal analyses and the pROC package used for ROC analyses. The robustness analyses were independently implemented in Python version 3.12.13 using NumPy version 2.3.5 and SciPy version 1.17.0. Normality was assessed using the Shapiro–Wilk test. Continuous variables were presented as mean ± standard deviation or median (interquartile range), and categorical variables as n (%). The primary outcome was analyzed using analysis of covariance (ANCOVA), with 60-min arterial pH as the dependent variable and device group as the main independent variable. The ANCOVA model was adjusted for baseline arterial pH, baseline PaCO_2_, baseline GCS score, baseline lactate level, age, COPD, and heart failure. Covariates were selected on clinical grounds as recognised determinants of early acid–base response, together with the baseline variables that showed meaningful imbalance between device groups (SMD≥0.20). Selection was therefore based on baseline distributions and clinical reasoning rather than on association with the outcome. The covariate set was fixed before the sensitivity analyses were performed and is identical across all models reported. Adjusted means, adjusted between-group differences, 95 % confidence intervals (CI), and p values were reported. To assess robustness to residual non-normality without changing the outcome, group contrast, or covariates, two sensitivity analyses were conducted: heteroskedasticity-consistent HC3 standard errors and a stratified nonparametric pairs bootstrap in which participants were resampled with replacement within device groups (20,000 resamples; fixed seed 20260826). A bias-corrected and accelerated (BCa) 95 % CI was calculated; the two-sided bootstrap p value was obtained from the null-centered empirical bootstrap distribution. Recorded inspiratory and expiratory pressure values were summarized as median (interquartile range) and compared using the Mann–Whitney U test. Other between-group comparisons used Student’s t-test or Mann–Whitney U test for continuous variables and chi-square or Fisher’s exact test for categorical variables, as appropriate. Repeated measurements between baseline and 60 min were analyzed using the Brunner–Langer nonparametric longitudinal approach, including group × time interaction analyses. Secondary physiological outcomes were exploratory, and their p values were interpreted descriptively because of the risk of multiplicity.

Factors associated with intubation were evaluated using multivariable logistic regression. Candidate variables were chosen on the basis of clinical relevance and previous literature on NPPV failure [12], 13], and were entered into the multivariable model if they were associated with intubation at p<0.10 in univariable analysis. Because final model membership was determined by this univariable screening, the resulting model is data-derived rather than prespecified. Multicollinearity among the final covariates was assessed using variance inflation factors (VIFs), with all VIFs<5 considered to indicate no problematic collinearity. Model fit was assessed using the Nagelkerke pseudo-R^2^ statistic, and calibration was assessed using the Hosmer–Lemeshow goodness-of-fit test. Discriminative performance was evaluated using receiver operating characteristic (ROC) analysis, and area under the curve (AUC) with 95 % CI was reported. All variables entered into these analyses were measured at 60 min after initiation of NPPV or represent the change between baseline and 60 min. They therefore characterise early response to treatment rather than baseline risk at presentation, and the analysis was not intended to derive a prediction model applicable at the time of ED arrival. All analyses were two-sided, and a p value<0.05 was considered statistically significant.

## Results

A total of 323 patients presenting to the ED with acute respiratory acidosis were screened. Before NPPV initiation and device selection, 97 patients were excluded: not meeting eligibility criteria (n=28), contraindications to NPPV (n=21), and immediate need for intubation (n=48). The remaining 226 patients were started on NPPV, 114 with cNPPV and 112 with Flow-Safe II+. After device selection, 22 patients were excluded from the analysis because of insufficient baseline or 60-min data (cNPPV: 14/114, 12.3 %; Flow-Safe II+: 8/112, 7.1 %). The analytic sample therefore comprised 204 patients: 100 in the cNPPV group and 104 in the Flow-Safe II+ group. During the study period, intubation occurred in 26 patients (cNPPV: n=15; Flow-Safe II+: n=11). The patient flow diagram is presented in Figure 2. Baseline balance was assessed using SMD. The groups were broadly similar in demographic characteristics, comorbidities, and most baseline physical examination findings. Most variables were well balanced; however, meaningful baseline imbalances were observed for GCS and selected blood gas parameters, particularly pH, HCO_3_^−^, lactate, and BE (Table 1).

**Figure 2:**
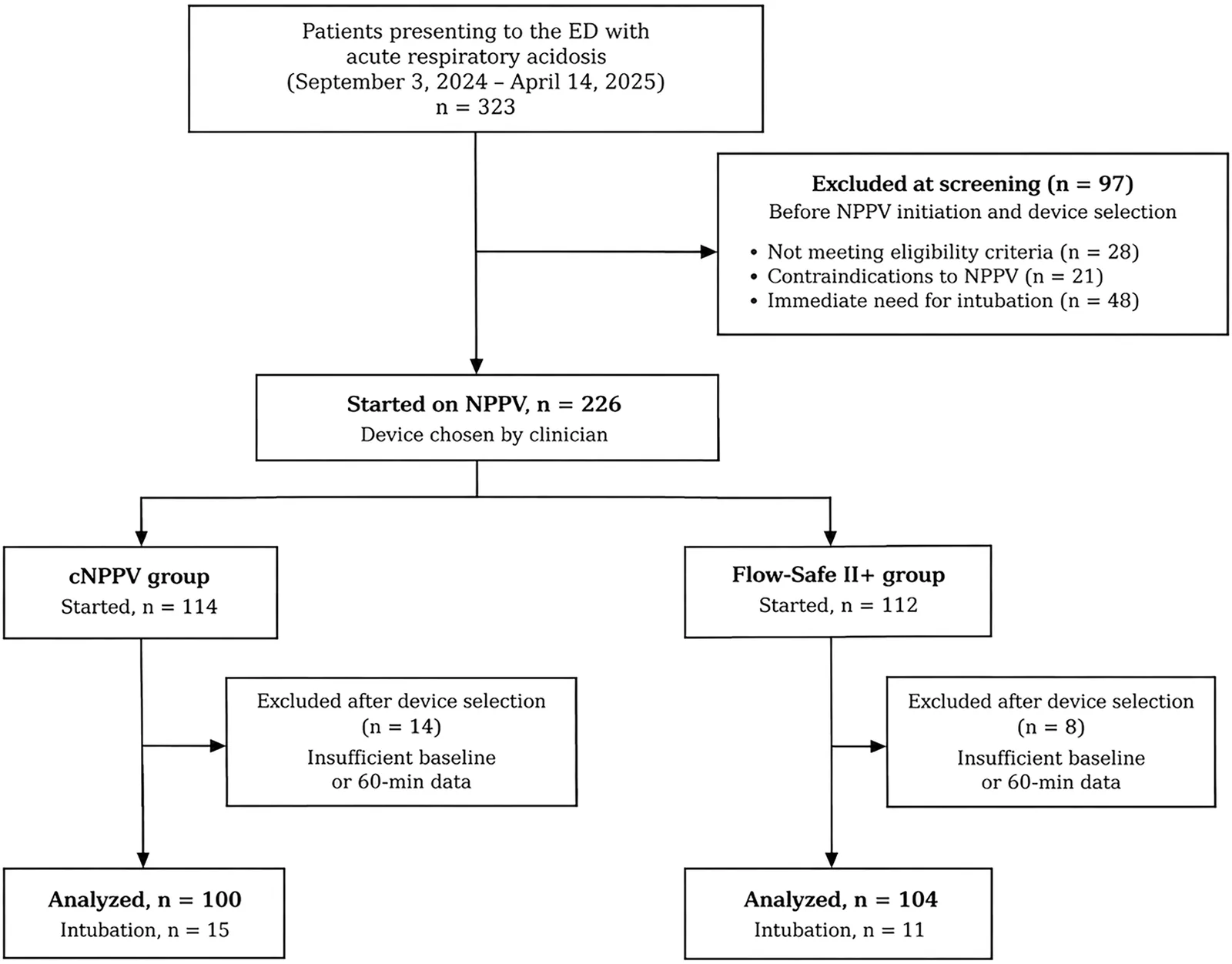
Patient flow diagram of the study. Of 323 patients screened, 97 were excluded before NPPV initiation and device selection, and 226 were started on NPPV with a device selected by the treating clinician. After device selection, 22 patients were excluded from the analysis because of insufficient baseline or 60-min data (14 in the cNPPV group and 8 in the Flow-Safe II+ group), leaving 204 patients in the analytic sample (100 in the cNPPV group and 104 in the Flow-Safe II+ group). Numbers of intubations during the study period are shown for each group. cNPPV, conventional noninvasive positive pressure ventilation; ED, emergency department; NPPV, noninvasive positive pressure ventilation.

**Table 1:** Baseline characteristics and clinical severity parameters of the study population.

	Total (n=204)	cNPPV (n=100)	Flow-Safe II+ (n=104)	SMD
Age, mean ± SD	72.34 ± 11.55	73.37 ± 12.04	71.34 ± 11.03	0.176
Female, n (%)	91 (44.6)	46 (46.0)	45 (43.3)	0.055
Hypertension	133 (65.2)	66 (66.0)	67 (64.4)	0.033
COPD	121 (59.3)	55 (55.0)	66 (63.5)	0.173
Heart failure	81 (39.7)	41 (41.0)	40 (38.5)	0.052
Diabetes mellitus	78 (38.2)	42 (42.0)	36 (34.6)	0.152
Coronary artery disease	68 (33.3)	34 (34.0)	34 (32.7)	0.028
Malignancy	37 (18.1)	17 (17.0)	20 (19.2)	0.058
Accessory respiratory muscle use	196 (96.1)	95 (95.0)	101 (97.1)	0.109
Tachypnea	193 (94.6)	96 (96.0)	97 (93.3)	0.121
Orthopnea	107 (52.5)	53 (53.0)	54 (51.9)	0.022
SBP (mmHg), mean ± SD	153.58 ± 36.05	151.19 ± 38.33	155.88 ± 33.73	0.130
DBP (mmHg), mean ± SD	85.97 ± 23.68	84.29 ± 24.12	87.58 ± 23.26	0.139
HR (beats/min), mean ± SD	107.08 ± 27.82	107.70 ± 29.73	106.49 ± 25.99	0.043
RR (breaths/min), mean ± SD	32.34 ± 5.77	32.50 ± 5.60	32.18 ± 5.96	0.055
SpO_2_ (%), median (IQR)	86 (8.5)	86 (7.7)	86 (9.0)	0.007
GCS score, median (IQR)	15 (10–15)	15 (10–15)	15 (12–15)	0.327
pH, mean ± SD	7.25 ± 0.07	7.24 ± 0.08	7.26 ± 0.05	0.335
PaCO_2_ (mmHg), mean ± SD	65.01 ± 11.93	64.4 ± 12.3	65.6 ± 11.6	0.103
HCO_3_^−^ (mmol/L), mean ± SD	24.71 ± 4.67	24.0 ± 5.2	25.4 ± 4.0	0.294
Lactate (mmol/L), mean ± SD	2.54 ± 2.20	2.9 ± 2.7	2.2 ± 1.5	0.317
BE (mmol/L), mean ± SD	0.26 ± 5.43	−0.3 ± 6.3	0.8 ± 4.4	0.206

Notably, the direction of these imbalances was consistent across variables: the Flow-Safe II+ group had higher baseline GCS, higher pH, higher HCO_3_^−^ and base excess, and lower lactate levels than the cNPPV group, indicating less severe baseline acidemia and overall less critical illness. This pattern suggests potential confounding by indication and was considered when interpreting between-group comparisons. SD, standard deviation; IQR, interquartile range; SMD, standardized mean difference; COPD, chronic obstructive pulmonary disease; SBP, systolic blood pressure; DBP, diastolic blood pressure; HR, heart rate; RR, respiratory rate; SpO_2_, peripheral oxygen saturation; GCS, Glasgow Coma Scale; PaCO_2_, partial pressure of carbon dioxide; HCO_3_^−^, bicarbonate; BE, base excess; cNPPV, conventional Noninvasive Positive Pressure Ventilation; Flow-Safe II+, Flow-Safe II+^®^ Disposable BiLEVEL/CPAP system.

In the adjusted ANCOVA model for the primary outcome, the adjusted mean 60-min arterial pH was 7.336 in the cNPPV group and 7.351 in the Flow-Safe II+ group. The adjusted between-group difference was 0.015 pH units (95 % CI, −0.006 to 0.036; p=0.159). The inference was unchanged with HC3 robust standard errors (adjusted difference, 0.015; 95 % CI, −0.006 to 0.036; p=0.165) and with the stratified BCa bootstrap (adjusted difference, 0.015; 95 % CI, −0.005 to 0.037; p=0.157), with all confidence intervals including zero (Table 2). Initial inspiratory and expiratory pressure settings were available for every participant. Recorded inspiratory pressure was lower in the Flow-Safe II+ group than in the cNPPV group [median 15 (IQR, 15–16) vs. 16 (IQR, 15–18) cmH_2_O; p<0.001], whereas recorded expiratory pressure was similar [5 (IQR, 5–6) vs. 5 (IQR, 5–6) cmH_2_O; p=0.469] (Supplementary Table S1). FiO_2_, oxygen flow rate, and ED arrival-to-NPPV initiation time were unavailable.

**Table 2:** ANCOVA model for the adjusted comparison of 60-min arterial pH between device groups.

Outcome/comparison	Adjusted estimate	95 % CI	p-Value
Adjusted mean 60-min arterial pH, cNPPV	7.336	7.321 to 7.351	–
Adjusted mean 60-min arterial pH, Flow-Safe II+	7.351	7.337 to 7.366	–
Adjusted difference, Flow-Safe II+ − cNPPV	0.015	−0.006 to 0.036	0.159
Sensitivity analysis: HC3 robust standard errors	0.015	−0.006 to 0.036	0.165
Sensitivity analysis: BCa bootstrap (20,000 resamples)	0.015	−0.005 to 0.037	0.157

The conventional ANCOVA, HC3 sensitivity analysis, and BCa-bootstrap sensitivity analysis used the same dependent variable (60-min arterial pH), device-group contrast, and covariates: baseline arterial pH, baseline PaCO_2_, baseline Glasgow Coma Scale score, baseline lactate, age, chronic obstructive pulmonary disease, and heart failure. The BCa interval used a stratified nonparametric pairs bootstrap with 20,000 within-group resamples; its p value was obtained from the null-centered empirical bootstrap distribution. ANCOVA, analysis of covariance; BCa, bias-corrected and accelerated; CI, confidence interval; cNPPV, conventional noninvasive positive pressure ventilation; COPD, chronic obstructive pulmonary disease; GCS, Glasgow Coma Scale; HC3, heteroskedasticity-consistent type 3 standard errors; PaCO_2_, partial pressure of carbon dioxide.

Repeated measurements between baseline and 60 min were evaluated as exploratory analysis using the Brunner–Langer nonparametric longitudinal approach. Most physiological parameters improved over time in both groups. In the group-by-time interaction analysis, pH and PaCO_2_ showed greater time-dependent improvement in the Flow-Safe II+ group compared with the cNPPV group (p for interaction=0.043 and 0.049, respectively). The interaction for SpO_2_ did not reach statistical significance, although a numerical trend toward greater improvement was observed in the Flow-Safe II+ group. No significant group-by-time interactions were observed for other physiological parameters (Table 3). Because these analyses were exploratory and were not adjusted for multiplicity, the corresponding p values are reported descriptively and should not be interpreted as confirmatory evidence of a treatment effect. No significant difference was observed between the groups in terms of intubation rate (cNPPV: 15.0 %, Flow-Safe II+: 10.6 %; p=0.344) or 24-h mortality (cNPPV: 1.0 %, Flow-Safe II+: 1.9 %; p=1.000) (Table 4).

**Table 3:** Changes in vital signs and blood gas parameters within the first 60 min of NPPV: longitudinal comparison between cNPPV and Flow-Safe II+ groups.

	cNPPV		Flow-Safe II+		p (time)	p (interaction)
	0 min	60 min	0 min	60 min		
**Blood gas parameters**						

pH, mean ± SD	7.24 ± 0.08	7.33 ± 0.09	7.26 ± 0.05	7.36 ± 0.07	<0.001	0.043
PaCO_2_ (mmHg), median (IQR)	60.6 (15.9)	49.1 (21.8)	62.7 (13.6)	47.3 (15.7)	<0.001	0.049
HCO_3_^−^ (mmol/L), mean ± SD	23.99 ± 5.21	25.05 ± 5.79	25.35 ± 3.95	26.37 ± 3.80	<0.001	0.159
BE (mmol/L), mean ± SD	−0.32 ± 6.25	0.32 ± 6.28	0.80 ± 4.39	1.22 ± 4.34	0.014	0.527
Lactate (mmol/L), median (IQR)	2.0 (2.27)	1.4 (1.20)	1.70 (1.77)	1.2 (0.87)	<0.001	0.463

**Vital signs and GCS**						

RR (breaths/min), mean ± SD	32.50 ± 5.60	23.22 ± 6.13	32.18 ± 5.96	22.94 ± 6.77	<0.001	0.800
SpO_2_ (%), median (IQR)	86 (7.7)	93 (4.0)	86 (9.0)	94 (4.0)	<0.001	0.084
HR (beats/min), mean ± SD	107.70 ± 29.73	96.58 ± 20.77	106.49 ± 25.99	96.15 ± 21.69	<0.001	0.607
SBP (mmHg), mean ± SD	151.19 ± 38.33	132.04 ± 25.04	155.88 ± 33.73	137.31 ± 22.37	<0.001	0.943
DBP (mmHg), mean ± SD	84.29 ± 24.12	73.50 ± 15.43	87.58 ± 23.26	76.78 ± 14.55	<0.001	0.855
GCS score, median (IQR)	15 (10–15)	15 (15–15)	15 (12–15)	15 (15–15)	0.026	0.188

Data are presented as mean ± standard deviation or median (interquartile range), according to distributional characteristics. Repeated measurements between baseline and 60 min were analyzed using the Brunner–Langer nonparametric longitudinal approach. The p (time) value indicates the overall time effect, whereas the p (interaction) value indicates the group-by-time interaction. Because multiple secondary physiological outcomes were evaluated, these analyses were considered exploratory and the corresponding p values were interpreted descriptively. SD, standard deviation; IQR, interquartile range; SBP, systolic blood pressure; DBP, diastolic blood pressure; HR, heart rate; RR, respiratory rate; SpO_2_, peripheral oxygen saturation; GCS, Glasgow Coma Scale; PaCO_2_, partial pressure of carbon dioxide; HCO_3_^−^, bicarbonate; BE, base excess; cNPPV, conventional Noninvasive Positive Pressure Ventilation; NPPV, Noninvasive Positive Pressure Ventilation.

**Table 4:** Clinical outcomes between the cNPPV and Flow-Safe II+ groups.

	cNPPV (n=100)	Flow-Safe II+ (n=104)	Total (n=204)	p-Value
**Clinical outcomes**				

Intubation rate, n (%)	15 (15.0)	11 (10.6)	26 (12.7)	0.344
Mortality within 24 h, n (%)	1 (1.0)	2 (1.9)	3 (1.5)	1.000

Data are presented as n (%). Group comparisons were performed using Pearson’s chi-square test or Fisher’s exact test when appropriate. The very small number of 24-h mortality events (n=3) precludes meaningful between-group comparison; therefore, this outcome is presented descriptively. The study was not formally powered for clinical endpoints, and the limited number of intubation events reduces the precision of the corresponding comparison. Longer-term mortality endpoints, including in-hospital and 30-day mortality, were not available. cNPPV, conventional noninvasive positive pressure ventilation.

In multivariable logistic regression analysis, the model demonstrated a Nagelkerke R^2^ of 0.545. The 60-min GCS score, 60-min SpO_2_ level, and the change in RR between baseline and 60 min were associated with intubation rate. Lower 60-min GCS and SpO_2_ values, along with a smaller improvement in RR, were associated with a higher intubation rate (Table 5). ROC analysis evaluated the discriminative performance of variables associated with intubation rate. The highest performance was observed for the change in RR between baseline and 60 min (AUC=0.809, 95 % CI: 0.730–0.888). Specifically, a reduction in respiratory rate of less than 5.5 breaths/min at 60 min (i.e., insufficient improvement in respiratory rate after initiation of NPPV) was associated with a higher intubation rate. Other variables with acceptable discrimination are presented in Table 6 and Figure 3.

**Table 5:** Multivariable logistic regression analysis of factors associated with intubation rate in patients receiving NPPV.

	β	SE	Wald	p-Value	OR (95 % CI)
Change in respiratory rate (0–60 min)	0.182	0.058	9.918	0.002	1.199 (1.071–1.342)
GCS score at 60 min	−1.173	0.286	16.781	<0.001	0.309 (0.176–0.542)
SpO_2_ at 60 min (%)	−0.234	0.111	4.400	0.036	0.792 (0.636–0.985)

Multivariable logistic regression analysis was performed to evaluate factors associated with intubation rate. The explanatory power of the model was Nagelkerke R^2^=0.545. p values are from the multivariable logistic regression model. NPPV, Noninvasive Positive Pressure Ventilation; CI, confidence interval; OR, odds ratio; β, beta coefficient; SE, standard error; Wald, Wald statistic; GCS, Glasgow Coma Scale; SpO_2_, peripheral oxygen saturation.

**Table 6:** Exploratory ROC curve analysis of clinical parameters associated with intubation rate in patients receiving NPPV.

	Cut-off value	Sensitivity (%)	Specificity (%)	p-Value	AUC	95 % CI
Change in respiratory rate	≥−5.5^a^	73.1	80.9	<0.001	0.809	0.730–0.888
GCS score at 60 min	≤14.5	65.4	92.7	<0.001	0.807	0.691–0.922
Respiratory rate at 60 min	≥23.0	88.5	64.0	<0.001	0.785	0.696–0.873
SpO_2_ at 60 min (%)	≤92.5	76.9	64.0	<0.001	0.722	0.613–0.832

Cut-off values were derived using the Youden index in the same cohort, which included only 26 intubation events, without internal or external validation. They are exploratory and should not be regarded as established clinical thresholds. The table presents clinical parameters with acceptable discriminative ability for intubation rate (AUC>0.70). p values are from the ROC curve analysis. ^a^Change in respiratory rate was calculated as the value at 60 min minus the value at baseline, so that negative values denote a reduction. A cut-off of ≥−5.5 breaths/min therefore corresponds to a reduction of less than 5.5 breaths/min, or an increase, and was associated with subsequent intubation. AUC, area under the curve; CI, confidence interval; GCS, Glasgow Coma Scale; SpO_2_, peripheral oxygen saturation.

**Figure 3:**
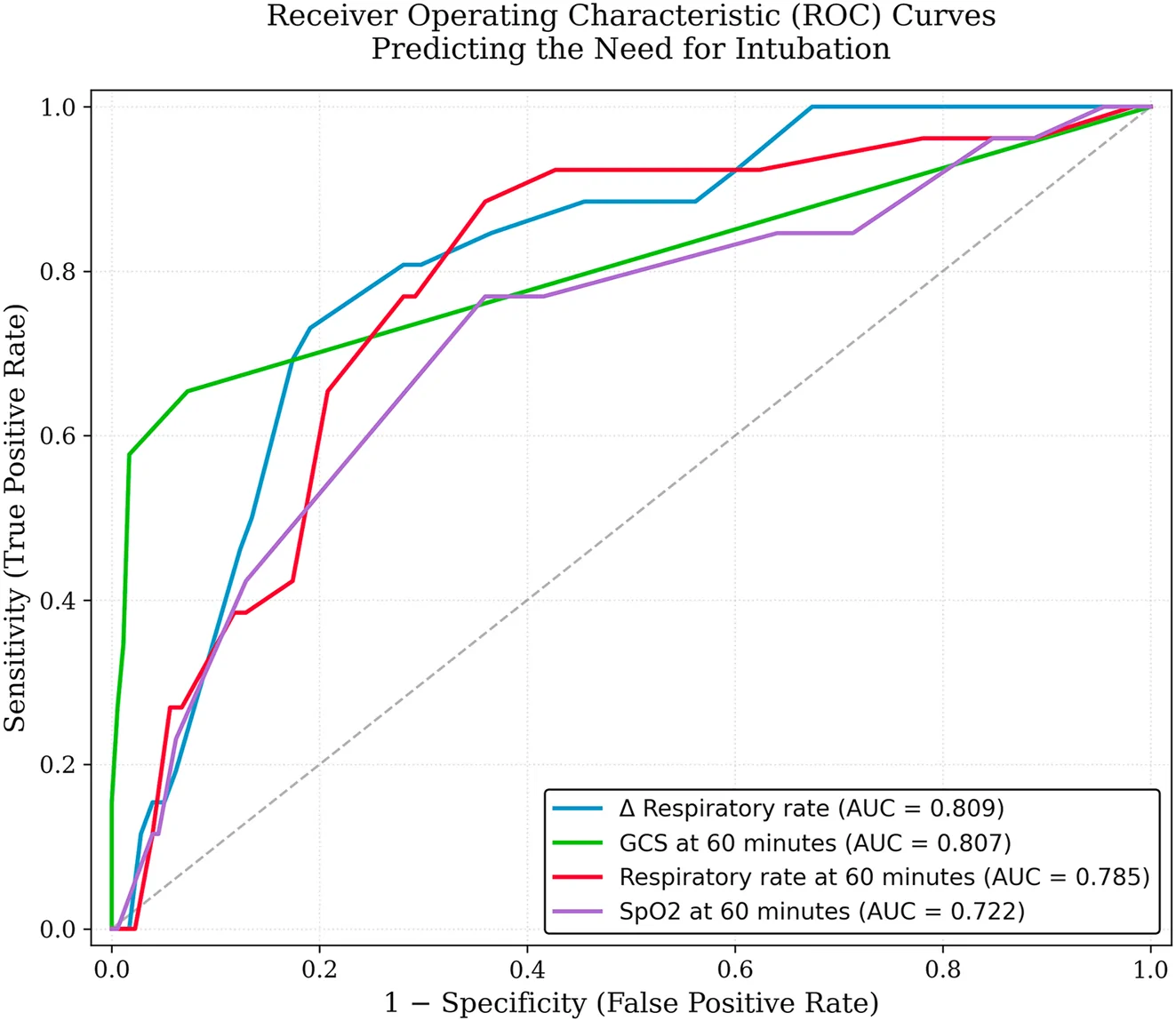
Receiver operating characteristic (ROC) curves of physiological variables measured during the first 60 min of noninvasive positive pressure ventilation, evaluated for their discriminative ability with respect to subsequent intubation. The change in respiratory rate between baseline and 60 min (Δ respiratory rate; blue), Glasgow Coma Scale at 60 min (green), respiratory rate at 60 min (red), and SpO_2_ at 60 min (purple) are shown. The diagonal dashed line represents the line of no discrimination (AUC=0.500). The cut-off values derived from these curves (Table 6) were obtained using the Youden index in the same cohort, which included only 26 intubation events, without internal or external validation; they are exploratory and should not be regarded as established clinical thresholds. AUC, area under the curve.

## Discussion

The principal finding of this prospective observational study is that, after adjustment for clinically selected baseline covariates, the adjusted 60-min arterial pH did not differ significantly between the Flow-Safe II+ and cNPPV groups (adjusted difference, 0.015 pH units; 95 % CI, −0.006 to 0.036; p=0.159). This null result for the primary endpoint should be considered the main result of our study. Intubation rate and 24-h mortality also did not differ significantly between groups, although the study was not formally powered for these clinical endpoints and the limited number of events precludes firm conclusions. The exploratory longitudinal Brunner–Langer analysis suggested a numerically greater time-dependent improvement in pH and PaCO_2_ in the Flow-Safe II+ group; however, given the non-randomized design, the observed baseline imbalances in variables related to disease severity (GCS, pH, HCO_3_^−^, lactate, and base excess), and the absence of multiplicity adjustment across multiple secondary outcomes, this longitudinal pattern should be regarded as hypothesis-generating rather than as evidence of device equivalence or superiority. Accordingly, these findings do not establish Flow-Safe II+ as equivalent or superior to conventional NPPV, and further randomized or rigorously controlled prospective studies are needed before definitive clinical recommendations can be made. An adjusted between-group difference of 0.015 pH units is also below the threshold typically considered clinically meaningful in acute respiratory acidosis, where changes of approximately 0.05–0.10 pH units are generally needed to reflect a clinically relevant treatment response. The absence of statistical significance is therefore consistent with the absence of a clinically meaningful between-device difference.

The adjusted primary result must nevertheless be read in the light of the baseline imbalances shown in Table 1. Although the groups were similar in age, sex, and major comorbidities, the Flow-Safe II+ group was systematically less severely ill at presentation, with higher GCS scores (SMD 0.33), higher arterial pH (SMD 0.34), higher HCO_3_^−^ (SMD 0.29) and base excess (SMD 0.21), and lower lactate concentrations (SMD 0.32). This pattern is consistent with confounding by indication, whereby clinicians preferentially selected the disposable device for patients perceived to be less critically ill. The ANCOVA model adjusted for baseline pH, PaCO_2_, GCS, and lactate, but covariate adjustment removes confounding only to the extent that the relevant dimensions of severity were measured and correctly specified. Residual confounding arising from unmeasured severity, clinician judgement, and underlying disease trajectory is therefore likely rather than merely possible, and the adjusted between-group difference should not be interpreted as an unbiased estimate of a device effect.

The direction of the imbalance is nevertheless informative. Because the less severely ill patients were preferentially treated with Flow-Safe II+, residual confounding would be expected to act in favour of that device. The adjusted difference already favours Flow-Safe II+ numerically (+0.015 pH units) while remaining non-significant and well below the 0.05–0.10 range regarded as clinically meaningful. A genuine benefit of the disposable system concealed by residual confounding is therefore not a plausible reading of these data, although a small advantage of conventional NPPV cannot be excluded. The same reasoning explains why the unadjusted longitudinal pattern of greater improvement in the Flow-Safe II+ group cannot be interpreted as a device effect. Significant improvements were observed in HR, SBP, DBP, RR, and SpO_2_ during the first 60 min in both groups. SpO_2_ improvement was numerically greater in the Flow-Safe II+ group (group × time interaction p=0.084), but, like the other interaction analyses, this result is exploratory and should not be interpreted as evidence of a true between-device difference in oxygenation. The overall improvement in oxygenation in both groups is consistent with the known physiological effects of positive airway pressure [14]. A previous study by our group in acute cardiogenic pulmonary oedema [9] reported comparable physiological improvement with a disposable CPAP system and cNPPV; however, that study addressed hypoxemic respiratory failure rather than hypercapnic respiratory acidosis, and findings are not directly extrapolable to the population studied here [9].

In the exploratory Brunner–Langer analysis, improvement in pH and PaCO_2_ was numerically greater in the Flow-Safe II+ group (unadjusted group × time interaction p=0.043 and p=0.049, respectively). Because several physiological outcomes were tested without adjustment for multiple comparisons, these p values are interpreted descriptively rather than as evidence of significant interactions. Previous studies on disposable CPAP systems have primarily focused on cardiogenic pulmonary edema or comparisons with standard medical therapy [15], 16]. Our study adds to the literature by directly comparing a disposable BiLEVEL system with cNPPV in patients with hypercapnic respiratory failure.

Whether the pH and PaCO_2_ interaction patterns reflect chance, device-related differences, or differences in treatment delivery cannot be determined from the present data. Device selection and ventilatory settings were determined by the treating clinicians as part of routine clinical care, without investigator intervention or a standardized study protocol. The available pressure data showed slightly lower recorded inspiratory pressure in the Flow-Safe II+ group and similar expiratory pressure between groups; however, these values represented the initial pressure settings only, and subsequent setting adjustments during the first 60 min were not systematically captured. Moreover, FiO_2_, oxygen flow rate, and the interval from ED arrival to NPPV initiation were not systematically recorded. Therefore, unmeasured differences in treatment delivery or timing cannot be excluded, and the pH and PaCO_2_ group × time interaction results should be interpreted as exploratory findings rather than evidence of a device-specific effect.

In logistic regression analysis, lower 60-min GCS score and SpO_2_ level, together with insufficient improvement in RR, were associated with a higher intubation rate, and the change in RR showed the highest discriminative performance in ROC analysis (AUC=0.809). It should be emphasised that every variable in these analyses was measured at 60 min or represents the change from baseline to 60 min. These findings therefore describe early response to treatment and are not baseline predictors: they cannot be applied at the moment of ED arrival, and the reported cut-off values are not triage thresholds. Their clinical relevance lies in structured reassessment at the end of the first hour of NPPV, at which point a reduction in respiratory rate of less than approximately 5.5 breaths/min, a depressed GCS, or a persistently low SpO_2_ identified patients who subsequently required intubation. This is consistent with previous work indicating that the trajectory of response during the first hours of noninvasive ventilation discriminates NPPV failure better than admission characteristics alone [12], 13]. Because these associations were derived and evaluated in the same cohort without external or internal validation, and because the number of intubation events was limited, the cut-off values should be regarded as descriptive rather than as validated decision thresholds. The baseline difference in GCS between device groups may additionally reflect clinician selection inherent to the observational design, and the GCS-related findings should accordingly be read in the context of baseline patient profile and early clinical trajectory rather than as a device effect.

Strengths: The present study has several strengths: a prospective design with consecutive patient screening to minimise recruitment-stage selection bias; an *a priori* sample-size calculation; a baseline-adjusted ANCOVA comparison supported by concordant HC3 and BCa-bootstrap sensitivity analyses; a clear distinction between the primary adjusted analysis and exploratory longitudinal analyses; and reporting consistent with the STROBE statement. The use of real-world emergency department data enhances external validity to comparable clinical settings.

Several important limitations must be considered when interpreting our findings. First, this was a single-center prospective observational study with non-randomized device allocation based on clinician discretion and on-site device availability. This design carries a substantial risk of selection bias and confounding by indication, and meaningful baseline imbalances were observed for variables directly related to disease severity and the principal outcome, including GCS score, baseline pH, HCO_3_^−^, lactate, and base excess. Although the ANCOVA adjusted for baseline pH, PaCO_2_, GCS, lactate, age, COPD, and heart failure, residual confounding due to unmeasured factors – such as clinician preference, patient-level workflow, and underlying disease trajectory – remains likely and cannot be quantified from these data. Second, ventilator settings were individualized by the treating clinicians as part of routine clinical care and were not standardized by the study investigators. Although initial inspiratory and expiratory pressure settings were available and are now reported, subsequent setting changes, FiO_2_, oxygen flow, mask interface, and time from ED arrival to NPPV initiation were not systematically captured. Therefore, the potential influence of differences in treatment delivery and timing on the observed early physiological responses could not be fully assessed. Third, the study focused on early physiological response and the in-ED clinical course; ICU admission and follow-up, in-hospital mortality, length of stay, return visits, and longer-term clinical outcomes were not evaluated. Fourth, the ANCOVA model showed residual non-normality, likely driven by extreme pH values. HC3 and BCa-bootstrap sensitivity analyses were concordant with the conventional ANCOVA and did not alter the inference, but the extreme observations remain relevant to precision. In addition, the limited number of intubation events reduced the statistical power of multivariable analyses, and multiple secondary physiological outcomes were tested without multiplicity adjustment. The multivariable and ROC analyses of intubation were based exclusively on variables measured at 60 min; no model predicting intubation from presentation characteristics was developed, and the reported cut-off values were derived and evaluated in the same cohort without independent validation. Fifth, blinding of clinicians and outcome assessors was not feasible. Finally, analyses were restricted to participants with complete baseline and 60-min data, with no imputation; 22 patients (14 cNPPV, 8 Flow-Safe II+) were excluded for this reason after device selection, and this complete-case approach may have introduced additional selection bias. In view of these limitations, the findings should be regarded as hypothesis-generating and should not be interpreted as evidence that disposable NPPV is equivalent or superior to conventional NPPV. Adequately powered randomized trials – or, where randomization is not feasible, rigorously designed multicenter observational studies applying propensity-score methods, standardized and serial ventilator-setting documentation, recorded treatment-timing data, and longer-term clinical endpoints – are required.

## Conclusions

In this prospective observational study of adults with acute hypercapnic respiratory acidosis managed in the emergency department, the adjusted 60-min arterial pH did not differ significantly between Flow-Safe II+ and cNPPV, and this inference was unchanged in HC3 and BCa-bootstrap sensitivity analyses. Intubation rate and 24-h mortality also did not differ significantly between groups, although the study was not powered for these clinical endpoints.

These findings should not be read as an efficacy comparison. Device allocation was determined by clinician judgement, and the resulting baseline imbalances in disease-severity variables were substantial and consistent in direction, with the disposable system preferentially used in less severely ill patients. Residual confounding is likely despite covariate adjustment, and the two cohorts are therefore not sufficiently comparable to support a reliable conclusion about the relative efficacy of the two devices. The greater time-dependent improvement observed in the Flow-Safe II+ group in exploratory longitudinal analyses is most plausibly attributable to this milder baseline disease state rather than to the device, and the recorded pressure values do not support a device-performance explanation. What these data support is narrower: in a real-world emergency department setting, a disposable oxygen-driven system was used to treat acute hypercapnic respiratory acidosis without a detectable difference in early arterial pH response, intubation rate, or 24-h mortality relative to conventional NPPV, in a comparison too confounded to establish equivalence, non-inferiority, or superiority. Adequately powered randomized trials – or, where randomization is not feasible, multicentre observational studies applying propensity-score methods with prospective documentation of ventilator settings and treatment timing and longer-term clinical endpoints – are required to determine the role of disposable NPPV in acute hypercapnic respiratory acidosis.

## Supplementary Material

Supplementary Material
