# Supplementary material for: Disposable vs. conventional NPPV in acute respiratory acidosis
Source: Open Med (Wars). 2026 Nov 2;21(1):20261563. doi: 10.1515/med-2026-1563 (PMC13726665; doi:10.1515/med-2026-1563)
Supplement: Supplementary file 1 [file j_med-2026-1563_suppl_001.docx]

**Supplementary Table S1.** Recorded ventilatory pressure values by device group

| **Recorded parameter** | **cNPPV (n = 100)** | | **Flow-Safe II+ (n = 104)** | | **p value** |
| --- | --- | --- | --- | --- | --- |
|  | **Median (IQR)** | **Mean ± SD** | **Median (IQR)** | **Mean ± SD** |  |
| Inspiratory pressure, cmH₂O | 16 (15–18) | 16.44 ± 1.95 | 15 (15–16) | 15.25 ± 1.53 | <0.001 |
| Expiratory pressure, cmH₂O | 5 (5–6) | 5.44 ± 0.86 | 5 (5–6) | 5.43 ± 1.07 | 0.469 |
| Pressure support, cmH₂O | 10 (10–12) | 11.00 ± 1.67 | 10 (9–10) | 9.82 ± 1.28 | <0.001 |

**Note:** Values are median (interquartile range) and mean ± standard deviation. P values are from two-sided Mann–Whitney U tests. The dataset contained the initial inspiratory and expiratory pressure settings at NPPV initiation for each participant; subsequent changes in settings during the first 60 minutes were not systematically captured. Pressure support is a derived variable, calculated as inspiratory minus expiratory pressure, and is reported as supplementary information. In the Flow-Safe II+ group, pressure levels were read from the integrated manometer and are therefore not directly equivalent to ventilator-displayed settings. FiO₂, oxygen flow rate, and the interval from ED arrival to NPPV initiation were not available.

**Abbreviations:** cmH₂O, centimetres of water; cNPPV, conventional noninvasive positive pressure ventilation; ED, emergency department; FiO₂, fraction of inspired oxygen; Flow-Safe II+, Flow-Safe II+® Disposable BiLEVEL/CPAP system; IQR, interquartile range; NPPV, noninvasive positive pressure ventilation; SD, standard deviation.
